# Analysis of energy-based algorithms for RNA secondary structure prediction

**DOI:** 10.1186/1471-2105-13-22

**Published:** 2012-02-01

**Authors:** Monir Hajiaghayi, Anne Condon, Holger H Hoos

**Affiliations:** 1 Computer Science Department, University of British Columbia, Vancouver, BC, Canada

## Abstract

**Background:**

RNA molecules play critical roles in the cells of organisms, including roles in gene regulation, catalysis, and synthesis of proteins. Since RNA function depends in large part on its folded structures, much effort has been invested in developing accurate methods for prediction of RNA secondary structure from the base sequence. Minimum free energy (MFE) predictions are widely used, based on nearest neighbor thermodynamic parameters of Mathews, Turner et al. or those of Andronescu et al. Some recently proposed alternatives that leverage partition function calculations find the structure with maximum expected accuracy (MEA) or pseudo-expected accuracy (pseudo-MEA) methods. Advances in prediction methods are typically benchmarked using sensitivity, positive predictive value and their harmonic mean, namely F-measure, on datasets of known reference structures. Since such benchmarks document progress in improving accuracy of computational prediction methods, it is important to understand how measures of accuracy vary as a function of the reference datasets and whether advances in algorithms or thermodynamic parameters yield statistically significant improvements. Our work advances such understanding for the MFE and (pseudo-)MEA-based methods, with respect to the latest datasets and energy parameters.

**Results:**

We present three main findings. First, using the bootstrap percentile method, we show that the average F-measure accuracy of the MFE and (pseudo-)MEA-based algorithms, as measured on our largest datasets with over 2000 RNAs from diverse families, is a reliable estimate (within a 2% range with high confidence) of the accuracy of a population of RNA molecules represented by this set. However, average accuracy on smaller classes of RNAs such as a class of 89 Group I introns used previously in benchmarking algorithm accuracy is not reliable enough to draw meaningful conclusions about the relative merits of the MFE and MEA-based algorithms. Second, on our large datasets, the algorithm with best overall accuracy is a pseudo MEA-based algorithm of Hamada et al. that uses a generalized centroid estimator of base pairs. However, between MFE and other MEA-based methods, there is no clear winner in the sense that the relative accuracy of the MFE versus MEA-based algorithms changes depending on the underlying energy parameters. Third, of the four parameter sets we considered, the best accuracy for the MFE-, MEA-based, and pseudo-MEA-based methods is 0.686, 0.680, and 0.711, respectively (on a scale from 0 to 1 with 1 meaning perfect structure predictions) and is obtained with a thermodynamic parameter set obtained by Andronescu et al. called BL* (named after the Boltzmann likelihood method by which the parameters were derived).

**Conclusions:**

Large datasets should be used to obtain reliable measures of the accuracy of RNA structure prediction algorithms, and average accuracies on specific classes (such as Group I introns and Transfer RNAs) should be interpreted with caution, considering the relatively small size of currently available datasets for such classes. The accuracy of the MEA-based methods is significantly higher when using the BL* parameter set of Andronescu et al. than when using the parameters of Mathews and Turner, and there is no significant difference between the accuracy of MEA-based methods and MFE when using the BL* parameters. The pseudo-MEA-based method of Hamada et al. with the BL* parameter set significantly outperforms all other MFE and MEA-based algorithms on our large data sets.

## Background

RNA molecules are essential to many functions in the cells of all organisms. For example, these molecules are involved in gene translation and also act as catalysts and as regulators of gene expression [1]. Because function is determined by molecular structure, there is significant investment in computational methods for predicting RNA secondary structure, which in turn is useful for inferring tertiary structure [2].

Our interest is in assessing the merits of some recent advances in secondary structure prediction in statistically robust ways. We focus on thermodynamically informed approaches for predicting pseudoknot free secondary structures from the base sequence. A widely used method finds the minimum free energy (MFE) structure with respect to the nearest neighbour thermodynamic model of Mathews, Turner and colleagues [3]. Some recent advances in secondary structure prediction are the new maximum expected accuracy (MEA-based) and maximum pseudo-expected accuracy (pseudo-MEA-based) methods of Lu et al. [4] and Hamada et al. [5,6]. These approaches generally maximize (pseudo) expected base pair accuracy as a function of base pair probabilities calculated using a partition function method and have higher average accuracy than the MFE algorithm on the Turner and Andronescu et al. energy parameters.

Knudsen et al. [7] and Do et al. [8] also presented some RNA secondary structure prediction methods based on probabilistic models of structures. But since their probabilistic approaches are not determined using a thermodynamic model, we don't include their methods in our later comparisons. Another advance is estimation of new energy parameters from both thermodynamic and structural data using state-of-the-art estimation techniques. Andronescu et al. [9] derived two parameter sets by inference from energies that were derived from optical melting experiments as well as from structural data. The two energy parameter sets are called BL* and CG*, named after the Boltzmann likelihood and constraint generation methods used to infer them. These parameter sets have yielded significant improvements in prediction accuracy of the MFE method, compared with the Turner parameters, with the BL* parameters being slightly better than the CG* parameters. Here and throughout, the accuracy of a prediction refers to its F-measure, which is the harmonic mean of sensitivity and positive predictive value (see Methods section for definitions of these measures). All of this work assesses algorithm accuracy on specific classes of RNAs, such as introns or transfer RNAs, as well as overall average accuracy on RNAs taken over all such classes.

This recent work motivates the following questions. Are comparisons of the (pseudo-)MEA-based and MFE approaches on specific RNA classes reliable when the size of available datasets is small? Do the MEA- or pseudo-MEA-based approaches produce significantly more accurate predictions than MFE on the latest energy parameter sets? What is the best combination of algorithm and thermodynamic model? To answer these questions, we report on the accuracy of both (pseudo-)MEA-based and MFE methods with respect to two versions of the Turner parameters as well as the recent BL* and CG* parameters of Andronescu et al., on datasets for specific RNA classes as well as large datasets that combine multiple RNA classes.

We present three main findings. First, we show that F-measure accuracies on our large datasets are likely to be reliable estimates of accuracy of a population represented by such sets, in the sense that high-confidence interval widths for F-measure obtained using the bootstrap percentile method are within a small, 2% range. Average accuracy on smaller classes is less reliable. For example, confidence intervals for both MEA and MFE have an 8% range on a class of 89 Group I introns that has been used previously in benchmarking algorithms. Second, there is a clear "winner" in terms of overall prediction accuracy, namely the pseudo-MEA-based method of Hamada et al. [6]. However, the relative accuracy of the MFE and MEA-based approaches depends on the underlying energy parameters: using a permutation test we find that, at a statistically significant level, the accuracy of MFE-based prediction on our large datasets is better on two of the four energy parameter sets that we consider, while MEA-based prediction is better than MFE-based prediction on a third parameter set. Finally, both MEA-based and MFE methods achieve the highest accuracy when using the fourth parameter set we consider, namely the BL* energy parameters of Andronescu et al. [9].

## Methods

In this section we first describe the datasets, thermodynamic models, and algorithms considered in this paper. We then describe the accuracy measures and statistical methods used in our analyses.

### Datasets

We use three datasets, as follows

• **S-Full **is a comprehensive set of 3,245 RNA sequences and their secondary structures that has been assembled from numerous reliable databases [10]. Sequences in this and our other datasets have length at most 700 nucleotides; in some cases these were derived by partitioning larger sequences such as 16S Ribosomal RNA sequences. The average length of sequences in S-Full is 270nt.

• **MT **was used by Lu et al. [4] in their study of the MEA algorithm and contains RNAs from the following classes: 16S Ribosomal RNA, 23S Ribosomal RNA, 5S Ribosomal RNA, Group I intron, Group II intron, Ribonuclease P RNA, Signal Recognition RNA and Transfer RNA.

• MA is a subset of the S-Full set, containing exactly those sequence of S-Full that are in the RNA classes included in MT. We formed the MA dataset in order to compare our algorithms on the same classes as did Lu et al., while using sets of RNAs from these classes with as large a size as possible.

An overview of different RNA classes in the MT and MA data sets is shown in Table 1. This table presents the number of RNA sequences, the mean and standard deviation of their lengths and the average of normalized similarity between RNA structures for each RNA class in these two data sets (see the later section on accuracy measures for a definition of normalized similarity).

**Table 1:** Overview of the different RNA classes in MT and MA data sets

RNA class	No. in MT	mean ± std of length	Avg. similarity	No. in MA	mean ± std of length	Avg. similarity
16S Ribosomal RNA	89	377.88 ± 167.18	0.60	675	485.66 ± 113.02	0.62
23S Ribosomal RNA	27	460.6 ± 151.3	0.53	159	453.44 ± 117.85	0.57
5S Ribosomal RNA	309	119.5 ± 2.69	0.88	128	120.98 ±3.21	0.88
Group I intron	16	344.88 ± 66.42	0.63	89	368.49 ±103.58	0.63
Group II intron	3	668.7 ± 70.92	0.70	2	578 ± 47	0.72
Ribonuclease P RNA	6	382.5 ± 41.66	0.74	399	332.78 ±52.34	0.72
Signal Recognition RNA	91	267.95 ± 61.72	0.71	364	227.04 ± 109.53	0.65
Transfer RNA	484	77.48 ± 4.8	0.96	489	77.19 ± 5.13	0.95

**Total**	1024			2305		

Overview of different RNA classes in the MT and MA data sets, including the number of RNAs, the mean and standard deviation of the length and the average normalized similarity between RNA sequences in these two data sets for each RNA class.

Lu et al. reported their results on a restricted format of the MT set in which certain bases of some sequences are in lower case, indicating that the base is unpaired in the reference structure. They have used this structural information in their predictions. However, we have not employed this information, and therefore our accuracy measures on the MT set are different from those of Lu et al. [4].

### Thermodynamic Models

A thermodynamic model consists of *features*--small structural motifs such as stacked pairs--plus *free energy change parameters*, one per feature. The first model we use, the Turner model [3] called "Turner99", is the most widely used energy model for prediction of RNA secondary structures. The model has over 7600 features, which are based on Turner's nearest neighbor rules and reflect the assumption that the stability of a base pair or loop depends on its sequence and on the adjacent base pair or unpaired bases. The model is additive in that the overall free energy change of a secondary structure for a given sequence is the sum of the free energy changes for features of the structure. The parameters of the model were derived from optical melting experiments, the most commonly used experimental approach to determine the free energy change of RNA structures.

We also consider variants of the Turner model, used by Andronescu et al. [9]. The T99-MultiRNAFold (T99-MRF) model is derived from the Turner99 model but includes only 363 features. Parameters for features in the Turner99 model can be obtained by extrapolation from the parameters of the T99-MRF model. Using maximum likelihood and constraint optimization methods, Andronescu et al. [9] derived new free energy change parameters for these 363 features; the resulting models are called BL* (for Boltzmann likelihood) and CG* (for constraint generation) respectively. We used all three models, namely T99-MRF, BL* and CG*, in this work, in order to assess the dependence of algorithm accuracy on model parameters. We note that in Lu et al.'s work [4], the parameters of a newer version of Turner model, called Turner2004, were used for one of the structural motifs, coaxial stacking. However, for the rest of structural motifs the parameters of Turner99 model were engaged. So, we also call the parameter set used for the Lu et al.'s benchmark Turner99 since most of its parameters are Turner99 ones and also the coaxial stacking motif is not employed in the other model that we study. Zakov et al. [11] also obtained parameters that improve RNA structure prediction. But we don't consider their parameters in this work, since those are not applicable for the partition function calculation and therefore for the probability calculation required for the MEA method.

### Algorithms

We analyze four RNA secondary structure prediction algorithms. The first predicts secondary structures that have minimum free energy (MFE) with respect to a given thermodynamic model. The second is the maximum expected accuracy (MEA) algorithm as introduced by Lu et al. [4], which maximizes expected base pair accuracy as a function of base pair probabilities calculated using a partition function method. We implemented the MEA algorithm for use with the MultiRNAFold models. As a result, we worked not only with the algorithms of Lu et al., which we refer to as rsMFE and rsMEA, but also with the MFE algorithm of Andronescu et al., referred to as ubcMFE, and a new implementation of MEA which we developed, called ubcMEA. Lu et al.'s benchmark [4] showed that rsMEA gives the best prediction accuracy when its γ parameter (which controls the relative sensitivity and positive predictive value) is equal to 1. Accordingly, we also set γ to be 1 in ubcMEA.

The third algorithm that we analyze is the generalized centroid estimator method of Hamada et al. [5]. This is similar to the MEA method, but uses a somewhat different objective function, namely a gamma-centroid estimator, to infer a structure from base pair probabilities. This method, which we refer to as gC-g1, employs a parameter γ to balance sensitivity and positive predictive value; based on the results of Hamada et al., we set γ = 1. The fourth method is another algorithm of Hamada et al. [6] that generalizes the centroid estimator by using a pseudo-expected accuracy maximization technique that automatically selects γ to balance sensitivity and positive predictive value for a given input. We refer to this algorithm as gC-pMFmeas (for generalized centroid maximized pseudo-expected accuracy).

The rsMEA and rsMFE algorithms always use the parameters of Turner99 model as their free energy parameter set, while ubcMEA and ubcMFE use parameter sets in the MultiRNAFold model format, namely the BL*, CG* and T99-MRF sets. The gC-g1 and gC-pMFmeas algorithms also employ BL* parameters using a Turner99 format.

### Accuracy Measures

We use three measures for determining the structural prediction accuracy, namely sensitivity (also called precision or precision rate), positive predictive value or PPV (also called recall), and F-measure, which combines the sensitivity and PPV into a single measure.

Sensitivity is the ratio of correctly predicted base pairs to the total base pairs in the reference structures. PPV is the fraction of correctly predicted base pairs, out of all predicted base pairs. F-measure is the harmonic mean of the sensitivity and PPV. This value is equal to the arithmetic mean when sensitivity and PPV are equal. However, F-measure becomes smaller than the arithmetic mean as one of the numbers approaches 0 (while the other is fixed). The possible values for these three measures are between 0 and 1; the closer to 1, the better prediction.

The F-measure is widely used measure in the literature; it is also the common measure in the studies by Hamada et al. and by Lu et al., to which refer in our study. Mostly to facilitate comparison of their results to ours, we decided to use the F-measure rather than the Matthews correlation coefficient (another well-known measure of accuracy predominantly used to assess binary classification methods).

$$\begin{array}{c} \text{sensitivity}=\frac{\text{number}\;\text{of}\;\text{correctly}\;\text{predicted}\;\text{base}\;\text{pairs}}{\text{number}\;\text{of}\;\text{base}\;\text{pairs}\;\text{in}\;\text{the}\;\text{reference}\;\text{structure}}\\ \text{PPV}=\frac{\text{number}\;\text{of}\;\text{correctly}\;\text{predicted}\;\text{base}\;\text{pairs}}{\text{number}\;\text{of}\;\text{predicted}\;\text{base}\;\text{pairs}}\\ \text{F - measure}=\frac{2\times \text{sensitivity}\times \text{PPV}}{\text{sensitivity}+\text{PPV}} \end{array}$$

Throughout this paper, we calculate three types of averages for a given measure "*M*" (which can be any of PPV, sensitivity, or F-measure), namely unweighted averages, weighted averages and S-weighted averages, defined below. The weighted average counts each sequence equally, regardless of which class it belongs to. The unweighted average, on the other hand, counts each class equally and was used by Lu et al [4]. A potential problem with the unweighted average is that an RNA class with many highly similar sequences can have disproportionate influence on the overall accuracy, relative to its sequence diversity. Therefore, we introduce the S-weighted average, which takes into account the similarities between RNA sequences in each RNA class and gives a weight to each one according to its average normalized similarity, in such a way that classes with highly similar sequences have lower weight.

The three averages are defined as follows when there are *n *RNA classes, *C*_1_*, C*_2_, ..., *C*_*n*_, with cardinalities *l*_1_,*l*_2_, ...,*l*_*n*_, respectively. For the remainder of our study, *n = *8, and the classes are those listed in Table 1).

(1) $$\begin{array}{c} \text{Unweighted}\;\text{Average}\;\text{of}\;M=\\ \frac{1}{n}\left(\frac{\sum_{\forall C_{1i}\in C_{1}}M\left(C_{1i}\right)}{l_{1}}+\frac{\sum_{\forall C_{2i}\in C_{2}}M\left(C_{2i}\right)}{l_{2}}+\cdots +\frac{\sum_{\forall C_{ni}\in C_{n}}M\left(C_{ni}\right)}{l_{n}}\right), \end{array}$$

(2) $$\begin{array}{c} \text{Weighted}\;\text{average}\;\text{of}\;M=\\ \frac{\sum_{\forall C_{1i}\in C_{1}}M\left(C_{1i}\right)+\sum_{\forall C_{2i}\in C_{2}}M\left(C_{2i}\right)+...+\sum_{\forall C_{ni}\in C_{n}}M\left(C_{ni}\right)}{l_{1}+l_{2}+...+l_{n}}, \end{array}$$

and

(3) $$\begin{array}{c} \text{S - Weighted}\;\text{average}\;\text{of}\;M=\\ \frac{l_{1}^{-s_{1}}\cdot \sum_{\forall C_{1i}\in C_{1}}M\left(C_{1i}\right)+l_{2}^{-s_{2}}\cdot \sum_{\forall C_{2i}\in C_{2}}M\left(C_{2i}\right)+...+l_{n}^{-s_{n}}\cdot \sum_{\forall C_{ni}\in C_{n}}M\left(C_{ni}\right)}{l_{1}^{1-s_{1}}+l_{{}_{2}}^{1-s_{2}}+...+l_{{}_{n}}^{1-s_{n}}}, \end{array}$$

where *s*_*i *_is the mean of the normalized similarities measured between the reference structures of any two of RNA sequences in the corresponding RNA class *C*_*i*_. Our normalized similarities were computed using the SimTree procedure by Eden et al. [12], which takes into account secondary structure similarities in addition to sequence similarities. The average normalized similarities are reported in Table 1 and always fall between zero and one, where an average normalized similarity of one means that all sequences in the set are identical. The S-weighted average equals the weighted average when *s*_*i *_is zero for all RNA classes, and it equals the unweighted average when *s*_*i *_is one for all classes. For the remaining cases, the value of the S-weighted average would fall between that of the unweighted and weighted averages.

### Bootstrap Percentile Confidence Intervals

We calculated the confidence intervals of average F-measure for various RNA classes, by using the bootstrap percentile method [13,14] following the recent work by Aghaeepour and Hoos [15]. We first took 10^4 ^resamples with replacement from the original F-measure values obtained by the prediction method under investigation on the given reference dataset, where all resamples had the same size as the original sample. We then computed the average F-measures of the resamples and employed these into the bootstrap distribution. Finally, we determined the lower and upper limits of the 95% bootstrap percentile confidence interval as the 2.5th and 97.5th percentile of the bootstrap distribution, respectively. These calculations were all performed using the "boot" package of the R statistics software environment [16].

We verified that the bootstrap distributions are close to Gaussian, using the Anderson-Darling test, which indicates that the bootstrap percentile intervals can be expected to be reasonably accurate [14].

### Permutation Test Method

To assess the statistical significance of the observed performance differences, we used permutation tests, following Aghaeepour and Hoos [15]. Since Lu et al. [4] and Hamada et al. [5] reported that the MEA-based methods outperform MFE, we used a one-sided permutation test [14] to determine whether MEA-based methods have significantly better accuracy than MFE on our parameter sets.

The test that we applied proceeds as follows. First, we calculated the difference in means between sets of F-measure values obtained by the two given structure prediction procedures, an MEA-based method and MFE. For simplicity, we call these two sets *A *and *B *and we denote their sizes as *n*_*A *_and *n*_*B*_, respectively. Then we combined the F-measure values of sets *A *and *B*. Next, we calculated and recorded the difference in sample means for 10^4 ^randomly chosen ways of dividing these combined values into two sets of size *n*_*A *_and *n*_*B*_. The p-value was then calculated as the proportion of the sample means thus determined whose difference was less than or equal to that of the means of sets *A *and *B*. Then, if the p-value is less than the 5% significance level, we reject the null hypothesis that MFE and the MEA-based method have equal accuracy and thus accept the alternative hypothesis that the MEA-based method has significantly better accuracy than MFE. Otherwise, we cannot reject the null hypothesis and therefore we cannot accept the alternative hypothesis.

Furthermore, to assess whether the difference in accuracy between the MEA-based methods and the MFE method on a given parameter set is significant, we performed a two-sided permutation test. This test works exactly like the one-sided permutation test, except that its p-value is calculated as the proportion of the sampled permutations where the absolute difference was greater than or equal to that of absolute difference of the means of sets *A *and *B*. Then, if the p-value of this test is less than the 5% significance level, we reject the null hypothesis that MFE and MEA have equal accuracy, otherwise, we cannot reject the null hypothesis.

All of these calculations were performed using the "perm" package of the R statistics software environment.

## Results and Discussion

In this section, we investigate to which degree the prediction accuracy of the energy-based methods studied in this paper is dependent on different datasets and different thermodynamic parameter sets used for prediction.

We start by considering how the size of a dataset will influence the accuracy achieved by RNA secondary structure prediction methods. We then study the dependency of prediction accuracy of the MFE, MEA, gC-g1, and gC-pMFmeas algorithms on the thermodynamic parameter sets that they use.

### Dependency of the Energy-based Methods on Data Characteristics

#### Results of the Energy-based Methods on Different Data Sets

Because measures of accuracy on reference datasets are used to assess the quality of prediction achieved by various algorithms, it is important to understand how such measures vary depending on the reference datasets used. Later in this section we also consider how accuracy measures vary depending on the energy model used.

The baseline data in Tables 2 and 3 show that there can be significant differences in accuracy of a given algorithm on RNA classes within the MA versus MT sets. For example, on Ribonuclease P RNA, when using the BL* parameter set, ubcMEA achieves an F-measure of 0.471 on the MT dataset (Table 2, fourth column, sixth row) and 0.643 on the MA set (Table 3, fourth column, sixth row), an absolute difference of about 17%.

**Table 2:** F-measure prediction accuracy of the MEA, MFE, gC-g1, and gC-pMFmeas algorithms on the MT dataset

RNA class	Class size	Mean ± std of length	F-meas (BL*)				F-meas (Turner99)	
			ubcMEA	ubcMFE	gC-g1	gC-pMFmeas	rsMEA	rsMFE
16S Ribosomal RNA	88	377.88 ± 167.18	0.649	0.621	0.640	0.659	0.574	0.539
23S Ribosomal RNA	27	460.6 ± 151.3	0.711	0.683	0.693	0.733	0.681	0.646
5S Ribosomal RNA	309	119.5 ± 2.69	0.739	0.743	0.725	0.746	0.625	0.642
Group I intron	16	344.88 ± 66.42	0.705	0.650	0.674	0.708	0.627	0.599
Group II intron	3	668.7 70.92	0.720	0.739	0.683	0.750	0.744	0.703
Ribonuclease P RNA	6	382.5 ± 41.66	0.471	0.460	0.519	0.495	0.517	0.522
Signal Recognition RNA	91	267.95 ± 61.72	0.641	0.621	0.633	0.637	0.518	0.557
Transfer RNA	484	77.48 ± 4.8	0.718	0.775	0.727	0.782	0.726	0.727

**Unweighted Average**			0.669	0.662	0.662	0.689	0.627	0.617
**Weighted Average**			0.710	0.732	0.707	0.743	0.660	0.665
**S-Weighted Average**			0.670	0.652	0.660	0.684	0.612	0.598

**Table 3:** F-measure prediction accuracy of the MEA, MFE, gC-g1, and gC-pMFmeas algorithms on the MA dataset

RNA class	Class size	Mean ± std of length	F-meas (BL*)				F-meas (Turner99)	
			ubcMEA	ubcMFE	gC-g1	gC-pMFmeas	rsMEA	rsMFE
16S Ribosomal RNA	675	485.66 ± 113.02	0.625	0.645	0.630	0.665	0.561	0.521
23S Ribosomal RNA	159	453.44 ± 117.85	0.645	0.643	0.626	0.664	0.588	0.562
5S Ribosomal RNA	128	120.98 ± 3.21	0.782	0.780	0.763	0.782	0.616	0.630
Group I intron	89	368.49 ± 103.58	0.644	0.631	0.642	0.670	0.576	0.550
Group II intron	2	578 ± 47	0.540	0.609	0.524	0.582	0.472	0.471
Ribonuclease P RNA	399	332.78 ± 52.34	0.643	0.603	0.656	0.678	0.615	0.575
Signal Recognition RNA	364	227.04 ± 109.53	0.730	0.721	0.680	0.708	0.609	0.625
Transfer RNA	489	77.19 ± 5.13	0.706	0.764	0.719	0.773	0.727	0.726

**Unweighted Average**			0.668	0.676	0.655	0.690	0.596	0.583
**Weighted Average**			0.672	0.682	0.669	0.708	0.618	0.600
**S-Weighted Average**			0.658	0.660	0.648	0.681	0.588	0.568

**Figure 1:**
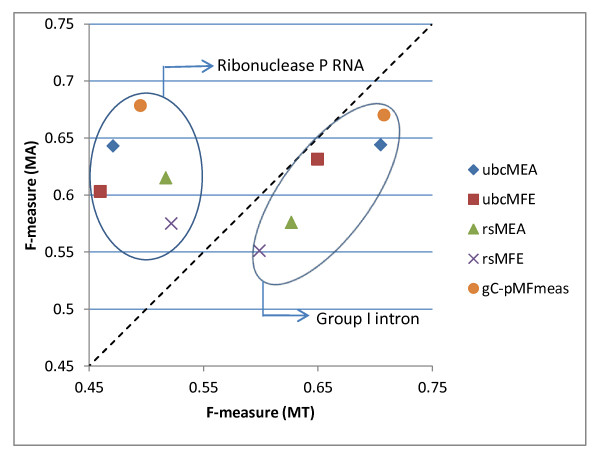
**Relative performance of the MEA and MFE algorithms on the MT and MA datasets for Ribonuclease P RNAs and Group I introns**.

Using data from Tables 2 and 3, Figure 1 illustrates the difference between accuracy measures on the MT versus MA datasets on both the Ribonuclease P RNA and the Group I intron classes, for the ubcMEA, ubcMFE and gC-pMFmeas algorithms, using the BL* parameters, as well as for rsMEA and rsMFE using the Turner99 parameters. In the figure, the further the points are from the dotted diagonal line, the larger the difference (algorithms for which there are no difference in average F-measure on the MT and MA sets would correspond to points on the main diagonal of the plot). Of the ten data points shown, only one represents a difference smaller than 3.2%. These data clearly indicate that, based only on prediction accuracy and without further statistical analysis, one cannot draw meaningful conclusions about the average accuracy of a particular algorithm on the overall population of an RNA class, based only on the average accuracy on currently available datasets.

#### Bootstrap Confidence Intervals for the Prediction Accuracy of the Energy-based Methods on different RNA Classes

Following the work of Aghaeepour and Hoos [15], we use bootstrap percentile confidence intervals to assess the accuracy of the average F-measures obtained by a secondary structure prediction procedure on a given RNA dataset; these provide a measure of the overall average accuracy on the whole population from which the dataset is drawn (see Methods for a detailed description of bootstrap percentiled confidence intervals). We chose to calculate confidence intervals using weighted average F-measure. We note from the data of Table 3 that on the MA dataset, all three averages (i.e., weighted, unweighted, and S-weighted) are qualitatively similar in the sense that if the F-measure accuracy of one algorithm is better than another with respect to one average, then the same is true with respect to the other averages. Thus, we would expect the same qualitative conclusions if we had used a different average.

**Figure 2:**
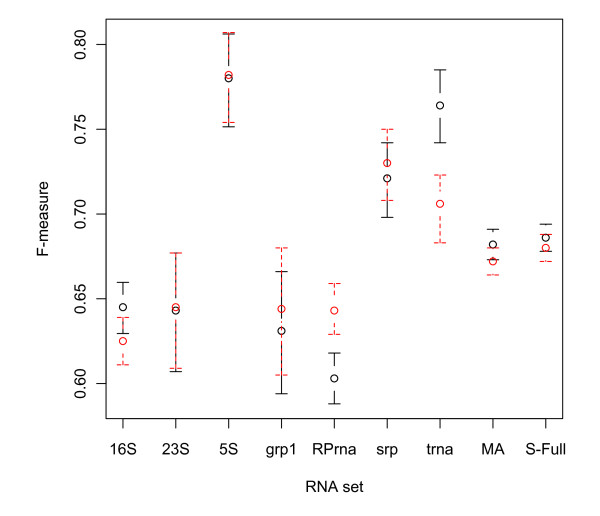
**95% bootstrap percentile confidence intervals for the F-measure average of the ubcMEA and ubcMFE algorithms**. 95% bootstrap percentile confidence intervals are shown for the F-measure average of the ubcMEA (dashed red bars) and ubcMFE (solid black bars) algorithms on the MA and S-Full sets and also different RNA classes in MA.

**Figure 3:**
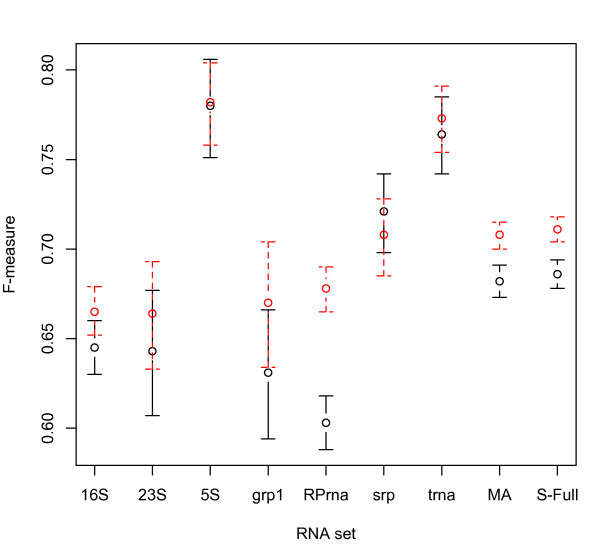
**95% bootstrap percentile confidence intervals for the F-measure average of the gC-pMFmeas and ubcMFE algorithms**. 95% bootstrap percentile confidence intervals are shown for the F-measure average of the gC-pMFmeas (dashed red bars) and ubcMFE (solid black bars) algorithms on the MA and S-Full sets and also different RNA classes in MA.

Figure 2 shows 95% bootstrap percentile confidence intervals for the ubcMEA and ubcMFE algorithms using the BL* parameter set, on individual RNA classes and on the MA and S-Full datasets. Figure 3 also indicates 95% bootstrap percentile confidence intervals for the ubcMFE and gC-pMFmeas algorithms using the BL* parameter set. Table 4 shows the confidence intervals for five algorithms, namely ubcMEA, ubcMFE, rsMEA, rsMFE and gC-pMFmeas. Because the Group II intron class contains only 2 RNAs, it had to be excluded from this analysis. Based on these results, we make several observations.

**Table 4:** Confidence intervals obtained by the bootstrap percentile method for the MA and S-Full sets and different RNA classes in MA

RNA class	Class size		Confidence Interval (CL = 0.95)			
		ubcMEA	ubcMFE	rsMEA	rsMFE	gC-pMFmeas
16S Ribosomal RNA	675	(0.611, 0.639)	(0.630, 0.660)	(0.548, 0.574)	(0.507, 0.536)	(0.652, 0.679)
23S Ribosomal RNA	159	(0.609, 0.677)	(0.607, 0.677)	(0.556, 0.618)	(0.530, 0.593)	(0.633, 0.693)
5S Ribosomal RNA	128	(0.754, 0.807)	(0.751, 0.806)	(0.573, 0.657)	(0.586, 0.671)	(0.758, 0.804)
Group I intron	89	(0.605, 0.680)	(0.594, 0.666)	(0.540, 0.611)	(0.513, 0.587)	(0.634, 0.704)
Ribonuclease P RNA	399	(0.629, 0.659)	(0.588, 0.618)	(0.602, 0.628)	(0.561, 0.588)	(0.665, 0.690)
Signal Recognition RNA	364	(0.708, 0.750)	(0.698, 0.742)	(0.583, 0.635)	(0.599, 0.651)	(0.685, 0.728)
Transfer RNA	489	(0.683, 0.723)	(0.742, 0.785)	(0.707, 0.747)	(0.705, 0.748)	(0.754, 0.791)

MA	2305	(0.664, 0.680)	(0.673, 0.691)	(0.610, 0.627)	(0.591, 0.609)	(0.700, 0.715)
S-Full	3246	(0.673, 0.688)	(0.678, 0.694)	(0.615, 0.631)	(0.598, 0.616)	(0.704, 0.718)

Confidence intervals are obtained by the bootstrap percentile method for the MA and S-Full sets and different RNA classes in MA (except Group II intron) when ubcMEA and ubcMFE use the BL* parameter set and rsMEA and rsMFE use the Turner99 parameter set and the confidence level (CL) is set to 0.95.

First, the confidence intervals of all algorithms on the MA and S-Full sets have a width of at most 0.018, indicating that the average accuracy measured on these sets is likely to be a good estimate - within 1% - of the accuracy of a population of RNA molecules represented by these sets. However, the interval widths on individual classes can much higher, e.g., 0.075 for ubcMEA on the Group I intron class, suggesting that average accuracy is not a reliable measure of a method's overall accuracy on such classes. We note that, without the use of rigorous statistical methods, a 0.02 difference in accuracy is considered significant in some related work [4].

Second, the confidence interval widths of RNA classes do not strictly decrease as the size of the class increases (i.e., the number of RNAs in the respective part of the reference set). For example, as shown in Table 4, for the ubcMEA (ubcMFE) algorithm the confidence interval width of the Ribonuclease P RNA class is 0.01 (0.013) less than the interval width for the Transfer RNA class, even though the Transfer RNA class contains roughly 1.2 times as many RNAs as are in the Ribonuclease P RNA class. Thus factors specific to the classes, other than class size, must account for confidence interval widths.

One possibility is that classes with greater similarity among structures would have smaller confidence intervals, since in the limit, if all sequences in a class are identical then the confidence interval has zero width. However, data from Tables 1 and 4 did not support such a correlation between average normalized similarity and confidence interval width, even for classes not too different in size. For example, the Ribonuclease P and Transfer RNA classes in the MA set have relatively similar sizes (399 and 489 sequences, respectively), with the Ribonuclease P RNA class having lower normalized similarity (0.72) than the Transfer RNA class (0.95), as one might expect; yet, for ubcMEA, the confidence interval for the Ribonuclease P RNA class is narrower (0.030) than those for the Ribonuclease P RNA class (0.040).

Understanding class-specific factors underlying differences in prediction accuracy remains a goal for further study.

**Figure 4:**
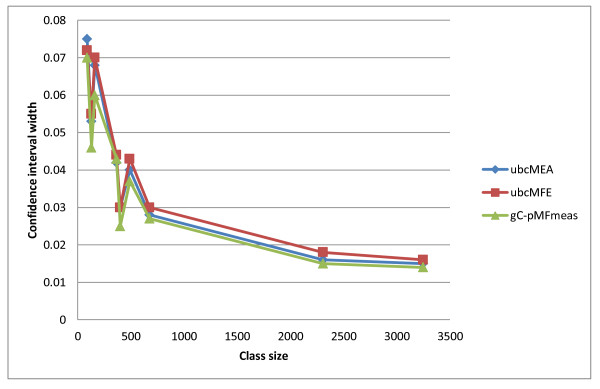
**Confidence interval width versus RNA class size in the MA set for the ubcMEA, ubcMFE and gC-pMFmeas methods**.

Third, for any given dataset (either a specific RNA class or the MA or S-Full sets), the confidence interval width for the ubcMFE algorithm is close to that for ubcMEA and gC-pMFmeas, even though the location of the interval (i.e., the prediction accuracy values) can be quite different. For example, the average F-measure of ubcMEA and ubcMFE on the Ribonuclease P RNA class differ by 0.04, but the confidence interval widths are identical. As another example, the smallest and largest confidence interval widths of the 16S Ribosomal RNA class among all four algorithms are, respectively, 0.026 and 0.030, which indicates a difference of less than 0.01 in the interval widths for this class (see Table 4). Figure 4 shows the width of confidence intervals versus the size of different RNA classes for the ubcMEA, ubcMFE and gC-PMFmeas methods. As one can see, almost all of the points for these three methods are coincident, indicating that ubcMEA, ubcMFE, and gC-PMFmeas have identical width in the corresponding sizes.

The other important observation from this figure is that, as one could expect, the width of the confidence interval tends to decrease with increasing number of RNAs of a given type in the reference set, but (as previously noted) there are notable exceptions to this trend for sets of size below 500.

The final interesting observation from Figure 3 and Table 4 is that the gC-pMFmeas method using the BL* parameter set outperforms all the other methods on most RNA classes and also on our large MA and S-Full sets. Hamada et al. [5] also showed that on a small set of 151 RNAs, gC-pMFmeas achieves better prediction accuracy than MFE and the other MEA-based methods when using the BL* parameter set. Our results confirm their finding on our large MA and S-Full data sets.

Similar graphs for the rsMEA and rsMFE algorithms are provided in the additional files. Additional files 1 and 2, Figures S4 and S5 support findings similar to those reported for ubcMEA, ubcMFE and gC-PMFmeas. Since the overall results on the gC-g1 algorithm were roughly the same as the results of the ubcMEA method, we report its prediction accuracies only in Tables 2 and 3 and did not study it further.

### Dependency of the Energy-based Methods on Thermodynamic Parameters

The accuracy of algorithmic methods achieved by energy-based secondary structure prediction approaches, such as gC-PMFmeas, MEA and MFE, depends on the underlying thermodynamic parameter sets. Lu et al. showed that the accuracy of MEA is better than that of MFE when using the Turner99 parameter set (on the MT reference set of RNAs). But does MEA or gC-PMFmeas outperform MFE on other published parameter sets? If so, is the difference in accuracy statistically significant? We address these questions in the following.

Table 5 presents prediction accuracy achieved for the various parameter sets on the S-Full dataset along with the associated 95% percentile confidence intervals. Additional file 3, Table S8 also shows similar results on S-Full-Test. Since the rsMEA and rsMFE algorithms use the Turner model, their prediction accuracies on the parameter sets of the MultiRNAFold model, which do not match the energy model underlying the rsMEA and rsMFE algorithms, cannot be obtained. Likewise, because the ubcMEA and ubcMFE algorithms use the MultiRNAFold model, their prediction accuracies on the parameter set of the Turner model cannot be obtained. We note that according to the results by Hamada et al., gC-pMFmeas achieves the highest prediction accuracies when using the BL* parameter set. Table 5 shows that there is no overlap between the confidence interval for gC-pMFmeas and those for the other algorithms, and that gC-pMFmeas has the highest average prediction accuracy.

**Table 5:** Accuracy comparison of different prediction algorithms with various parameter sets on the S-Full set

Algorithm	F-Measure			
	T99-MRF	BL*	CG*	Turner99
**ubcMEA**	0.582 (0.574,0.591)	**0.680 **(0.673,0.688)	0.636 (0.628,0.644)	n/a
**ubcMFE**	0.601 (0.592,0.609)	**0.686 **(0.678,0.694)	0.671 (0.663,0.679)	n/a

**rsMEA**	n/a	n/a	n/a	0.623 (0.615,0.632)
**rsMFE**	n/a	n/a	n/a	0.607 (0.598,0.615)

**gC-pMFmeas**	-	**0.711 **(0.704, 0.718)	-	-

The table presents the prediction accuracy of different algorithms with different thermodynamic sets in terms of F-measure. The 95% percentile confidence intervals of their accuracies are also shown in parentheses. The parameter set T99-MRF refers to the Turner99 parameters in MultiRNAFold format. BL* and CG* are the parameter sets obtained by the BL and CG approaches of Andronescu et al. [9], respectively. Also, the Turner99 parameter set is the parameter set obtained by Mathews et al. [3]. "n/a" indicates cases in which a given algorithm is not applicable to a parameter set, since that does not match the energy model underlying the algorithm. The highest accuracies for MEA and MFE are shown in bold.

In contrast, the relative accuracy of MEA versus MFE varies, with MFE being slightly better than MEA in two cases, MEA being slightly better than MFE in one case, and both having approximately the same accuracy in one case, namely BL*. To assess whether MEA has significantly better accuracy than MFE on our parameter sets, we first performed the one-sided permutation test (see Methods) with the alternative hypothesis that MEA yields higher prediction accuracy than MFE. As recorded in Table 6, the resulting p-value falls below the standard significance threshold of 0.05 only for Turner99, indicating that only for this parameter set, MEA outperforms MFE, and its performance, only on this parameter set, can be considered better than MFE. In contrast, the p-values obtained for the other parameter sets, namely CG*, T99-MRF and BL* are above 0.05, indicating that we cannot conclude that MEA outperforms MFE for these parameters sets.

**Table 6:** P-value of one-sided permutation test for the MEA versus MFE algorithm on different parameter sets

Parameter set	T99-MRF	BL*	CG*	Turner99
**p-value**	0.999	0.848	1	**0.003**

P-value of one-sided permutation test is computed for the MEA versus MFE algorithm on different parameter sets; p-values below the standard significance level of 0.05 are shown in bold face and indicate significantly higher prediction accuracy of MEA compared to MFE.

We next performed the two-sided permutation test on all mentioned parameter sets. The results, as recorded in Table 7, indicate that for the BL* parameter set, the performance difference between MFE and MEA is not statistically significant (at the standard significance threshold of 0.05), while the performance differences for the other two parameter sets, CG* and T99-MRF, are statistically significant; we note that in both cases, MFE achieved higher prediction accuracy than MEA and their confidence intervals don't overlap (see Table 5).

**Table 7:** P-value of two-sided permutation test for the MEA and MFE algorithms on different parameter sets

Parameter set	T99-MRF	BL*	CG*	Turner99
**p-value**	**0.003**	0.299	**0**	**0.006**

P-value of two-sided permutation test is computed for the MEA and MFE algorithms on different parameter sets; p-values below the standard significance level of 0.05 are shown in bold face and indicate significant differences in prediction accuracy.

Furthermore, the results in Table 5 show that of the parameter sets we considered, BL* gives the highest prediction accuracy, regardless of whether the MEA or MFE prediction algorithm is used. This is consistent with earlier results by Andronescu et al. [9] regarding the performance of ubcMFE using the BL* parameter set.

Finally, we observe that for a given class of RNAs, depending on the energy model, MFE sometimes performs better than MEA and vice versa (see Table 4 and Figure 2). For example, when considering our set of 16S Ribosomal RNAs and using the BL* parameter set, MFE outperforms MEA by 0.02, while for the Full Turner 99 parameters, MEA outperforms MFE by 0.04 (both performance differences are statistically significant based on a permutation test with significance threshold 0.05).

Overall, we conclude that the relative performance of MEA versus MFE depends on the thermodynamic parameter set used, and that both ubcMEA and ubcMFE with the BL* parameter set have significantly better accuracy than rsMEA with the Turner99 parameter set.

## Conclusions

Improvements both in algorithmic methods and in thermodynamic models lead to important advances in secondary structure prediction. In this work, we showed that gC-pMFmeas with the BL* parameter set significantly outperforms the other MFE and MEA-based methods we studied. However, the relative performance of MEA-based and MFE methods vary depending on the thermodynamic parameter set used. For example, MEA-based methods significantly outperform minimum free energy (MFE) methods for the Turner99 model but the opposite is true for other models and in fact the difference in performance between MEA-based and MFE methods is negligible on our best thermodynamic model, BL*. Our findings suggest that, as thermodynamic models continue to evolve, a diverse toolbox of algorithmic methods will continue to be important.

We also showed the importance of using large datasets when assessing accuracy of specific algorithms and thermodynamic models. Specifically, we showed that bootstrap percentile confidence intervals for average prediction accuracy on our two largest datasets, MA and S-Full, have narrow widths (at most 0.018), indicating that the average accuracies measured on these sets are likely to be good estimates of the accuracies of the populations of RNA molecules they represent. In contrast, interval widths for several of the specific RNA classes studied in the paper were much larger, with no clear correlation between confidence interval width and either class size or average normalized similarity. It may be the case that confidence interval widths are correlated with the distribution of evolutionary distances among the class members, rather than on the more simplistic average normalized similarity that we consider in this paper; studying this further would be an interesting direction for future research. Regardless, our analysis shows that larger datasets are needed to obtain reliable accuracy estimates on specific classes of RNAs, underscoring the importance of continued expansion of RNA secondary structure data sets.

Our work illustrates the use of statistical techniques to gauge the reliability and significance of measured accuracies of RNA secondary structure prediction algorithms. We hope that this work will provide a useful basis for ongoing assessment of the merits of RNA secondary structure prediction algorithms.

## Authors' contributions

MH acquired, analyzed and interpreted the data and drafted the manuscript. AC supervised the research and participated in analysis of data and revising the manuscript. HH consulted in approaches used and participated in data interpretation and manuscript revision. All authors read and approved the final manuscript.

## Supplementary Material

Additional file 1 **95% bootstrap percentile confidence interval graphs for the F-measure average of the rsMEA and rsMFE**.95% bootstrap percentile confidence intervals are shown for the F-measure average of the rsMEA (dashed red bars), and rsMFE (solid black bars) algorithms on the MA and S-Full sets and also different RNA classes in MA. Click here for file

Additional file 2 **Confidence interval width versus RNA class size in the MA set for the rsMEA and rsMFE methods**. The figure shows the confidence interval width of RNA classes in the MA set for the rsMEA and rsMFE methods. Click here for file

Additional file 3 **Accuracy comparison of different prediction algorithms with various parameter sets on the S-Full-Test set**. The table presents the prediction accuracy of different algorithms with different thermodynamic sets in terms of F-measure. The parameter set T99-MRF refers to the Turner99 parameters in MultiRNAFold format. BL* and CG* are the parameter sets obtained by the BL and CG approaches of Andronescu et al. [9], respectively. Also, the Turner99 parameter set is the parameter set obtained by Mathews et al. [3]. "n/a" indicates cases in which a given algorithm is not applicable to a parameter set, since that does not match the energy model underlying the algorithm. The highest accuracies for MEA and MFE are shown in bold. Click here for file
