# Supplementary figures and images for: Analysis of energy-based algorithms for RNA secondary structure prediction
Source: BMC Bioinformatics. 2012 Feb 1;13:22. doi: 10.1186/1471-2105-13-22 (PMC3347993; doi:10.1186/1471-2105-13-22)

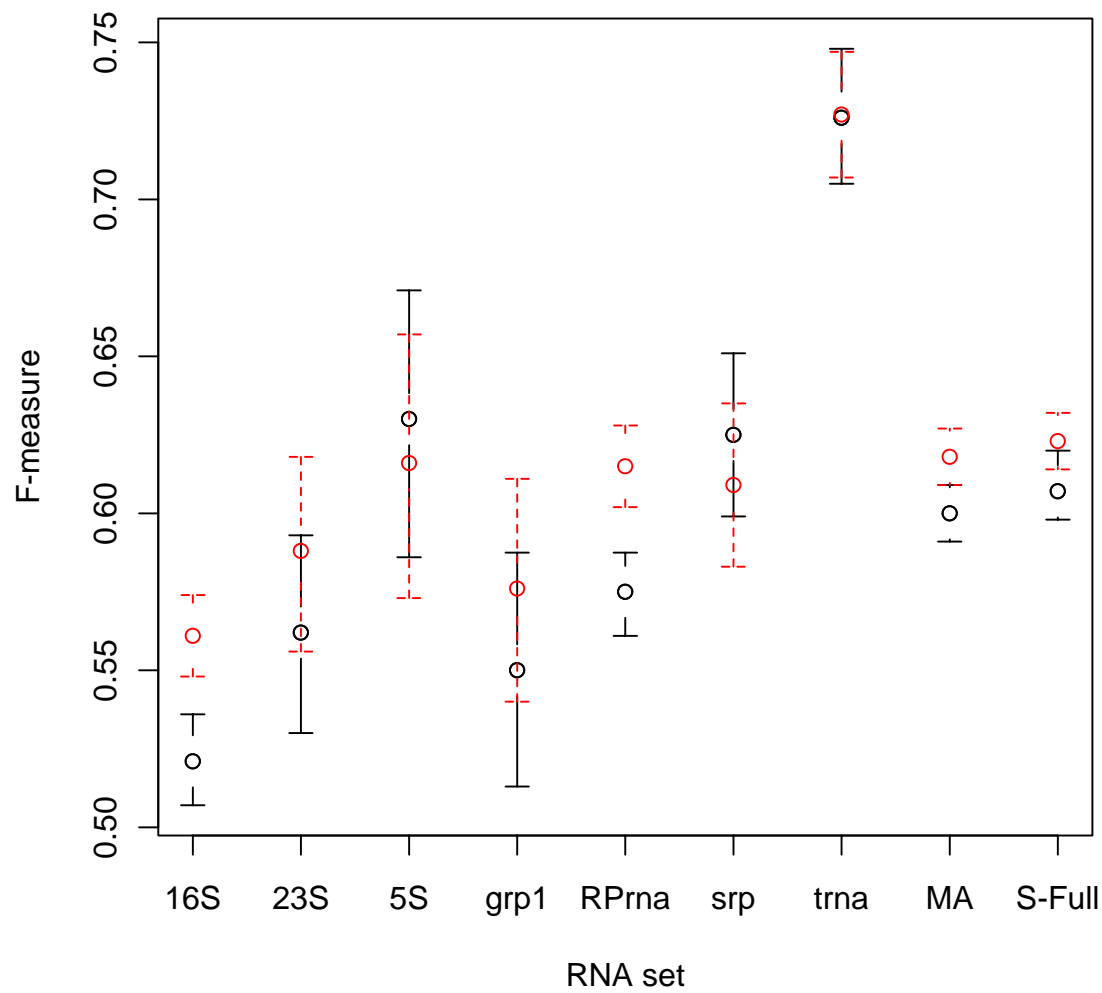

Supplement: Additional file 1 — 95% bootstrap percentile confidence interval graphs for the F-measure average of the rsMEA and rsMFE.95% bootstrap percentile confidence intervals are shown for the F-measure average of the rsMEA (dashed red bars), and rsMFE (solid black bars) algorithms on the MA and S-Full sets and also different RNA classes in MA. [file 1471-2105-13-22-S1.PDF]

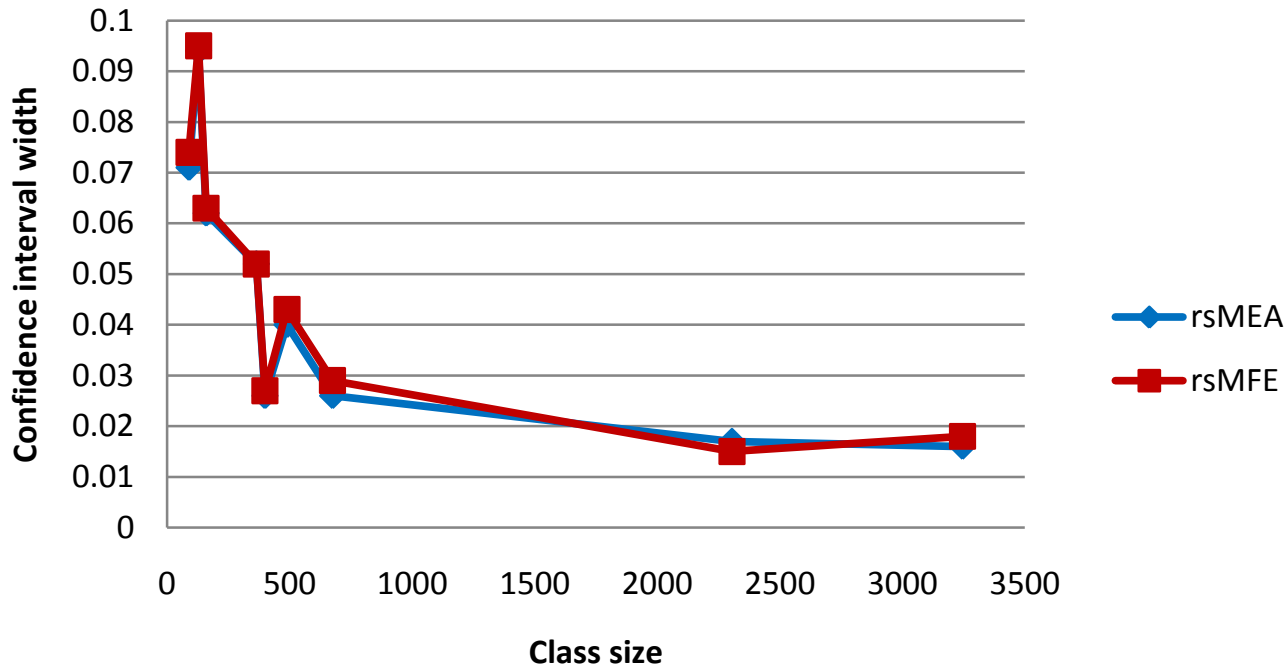

Supplement: Additional file 2 — Confidence interval width versus RNA class size in the MA set for the rsMEA and rsMFE methods. The figure shows the confidence interval width of RNA classes in the MA set for the rsMEA and rsMFE methods. [file 1471-2105-13-22-S2.PDF]
