# Supplementary material for: Analysis of energy-based algorithms for RNA secondary structure prediction
Source: BMC Bioinformatics. 2012 Feb 1;13:22. doi: 10.1186/1471-2105-13-22 (PMC3347993; doi:10.1186/1471-2105-13-22)
Supplement: Additional file 3 — Accuracy comparison of different prediction algorithms with various parameter sets on the S-Full-Test set. The table presents the prediction accuracy of different algorithms with different thermodynamic sets in terms of F-measure. The parameter set T99-MRF refers to the Turner99 parameters in MultiRNAFold format. BL* and CG* are the parameter sets obtained by the BL and CG approaches of Andronescu et al. [9], respectively. Also, the Turner99 parameter set is the parameter set obtained by Mathews et al. [3]. "n/a" indicates cases in which a given algorithm is not applicable to a parameter set, since that does not match the energy model underlying the algorithm. The highest accuracies for MEA and MFE are shown in bold. [file 1471-2105-13-22-S3.PDF]

Algorithm	F-Measure			
	T99-MRF	BL*	CG*	Turner99
**ubcMEA**	0.571	**0.675**	0.636	n/a
**ubcMFE**	0.597	**0.672**	0.669	n/a
**rsMEA**	n/a	n/a	n/a	0.616
**rsMFE**	n/a	n/a	n/a	0.600
**gC-pMFmeas**	-	**0.700**	-	-
